# Assessments at multiple levels of biological organization allow for an integrative determination of physiological tolerances to turbidity in an endangered fish species

**DOI:** 10.1093/conphys/cow004

**Published:** 2016-03-16

**Authors:** Matthias Hasenbein, Nann A Fangue, Juergen Geist, Lisa M Komoroske, Jennifer Truong, Rina McPherson, Richard E Connon

**Affiliations:** af1 Department of Anatomy, Physiology and Cell Biology, School of Veterinary Medicine, University of California, Davis, CA 95616, USA; af2 Department of Wildlife, Fish & Conservation Biology, University of California, Davis, CA 95616, USA; af3 Chair of Aquatic Systems Biology, Department of Ecology and Ecosystem Management, Technische Universität München, Mühlenweg 22, Freising D-85354, Germany

**Keywords:** Delta smelt, fundamental niche, habitat preference, *Hypomesus transpacificus*, stress

## Abstract

Turbidity can influence trophic levels by altering species composition and can potentially affect fish feeding strategies and predator–prey interactions. The estuarine turbidity maximum, described as an area of increased suspended particles, phytoplankton and zooplankton, generally represents a zone with higher turbidity and enhanced food sources important for successful feeding and growth in many fish species. The delta smelt (*Hypomesus transpacificus*) is an endangered, pelagic fish species endemic to the San Francisco Estuary and Sacramento–San Joaquin River Delta, USA, where it is associated with turbid waters. Turbidity is known to play an important role for the completion of the species' life cycle; however, turbidity ranges in the Delta are broad, and specific requirements for this fish species are still unknown. To evaluate turbidity requirements for early life stages, late-larval delta smelt were maintained at environmentally relevant turbidity levels ranging from 5 to 250 nephelometric turbidity units (NTU) for 24 h, after which a combination of physiological endpoints (molecular biomarkers and cortisol), behavioural indices (feeding) and whole-organism measures (survival) were determined. All endpoints delivered consistent results and identified turbidities between 25 and 80 NTU as preferential. Delta smelt survival rates were highest between 12 and 80 NTU and feeding rates were highest between 25 and 80 NTU. Cortisol levels indicated minimal stress between 35 and 80 NTU and were elevated at low turbidities (5, 12 and 25 NTU). Expression of stress-related genes indicated significant responses for *gst*, *hsp70* and *glut2* in high turbidities (250 NTU), and principal component analysis on all measured genes revealed a clustering of 25, 35, 50 and 80 NTU separating the medium-turbidity treatments from low- and high-turbidity treatments. Taken together, these data demonstrate that turbidity levels that are either too low or too high affect delta smelt physiological performance, causing significant effects on overall stress, food intake and mortality. They also highlight the need for turbidity to be considered in habitat and water management decisions.

## Teaser

This study evaluates turbidity requirements of an endangered fish species using physiological endpoints at multiple levels of biological organization, in particular whole body cortisol, molecular biomarkers, survival, and feeding response. The importance of turbidity and the need for an understanding of the mechanistic behind the observed physiological response are highlighted.

## Introduction

Estuaries are among the most threatened, anthropogenically modified and managed ecosystems worldwide (Kennish, 2002, Lotze *et al.*, 2006). This is certainly the case in the San Francisco Estuary (herein referred to as ‘Estuary’) and Sacramento–San Joaquin River Delta (herein referred to as ‘Delta’), USA, representing a paragon for such heavily influenced ecosystems (Nichols *et al.*, 1986). Estuaries are a unique ecosystem type denoted by the interface between coastal marine and riverine freshwater habitats (Wołowicz *et al.*, 2007) and provide a dynamic habitat for highly specialized and adapted species. Characteristic of estuaries are mixing zones, which originate from freshwater outflow, saltwater intrusion and a distinctive recurring ebb tide–flood scenario. In these zones, environmental factors such as temperature, salinity, turbidity and flow can change quickly, posing additional challenges for organisms.

Environmental factors, whether abiotic or biotic, that cause stress in an organism evoke a stress response at multiple levels of biological organization, including the level of molecules, cells, organs, organ systems and organisms (Ricklefs and Wikelski, 2002; Kassahn *et al.*, 2009; Schulte *et al.*, 2011; Schulte, 2014). These physiological stress parameters can be used as proxies for fitness and performance, leading to performance curves that have been used to describe tolerances and niche dimensions (Shelford, 1931; Pörtner *et al.*, 2010; Schulte, 2014). Precise information on tolerances and niche dimensions of a species help understand its distribution, which is one of the fundamental goals of ecology and physiology.

Stress is often described as a state of threatened or disturbed homeostasis, caused by either internal or external stressors, which can be restored by a complex repertoire of adaptations comprising physiological and behavioural responses (Chrousos and Gold, 1992; Chrousos, 1998; Schulte, 2014). The physiological stress responses in fish can be categorized as primary, secondary and tertiary stress responses (Iwama, 1998; Barton, 2002; Wendelaar Bonga, 2011). The primary stress response is characterized by immediate endocrine changes in stress hormones such as catecholamines, adrenocorticotrophic hormone and cortisol (Iwama, 1998; Barton, 2002; Wendelaar Bonga, 2011). The secondary response is described by the physiological effects of the primary stress hormones, in particular the activation of metabolic pathways, hydromineral balance, immune and respiratory functions (Iwama, 1998; Barton, 2002; Wendelaar Bonga, 2011). The tertiary response encompasses changes in behaviour and physiology observed at the whole-animal level, such as growth, development, reproduction, disease resistance and survival (Iwama, 1998; Barton, 2002; Wendelaar Bonga, 2011). Recent reviews have highlighted the need for a mechanistic understanding of physiological responses of fish to environmental stressors for effective fisheries management and optimal conservation efforts (Geist, 2015; Horodysky *et al.*, 2015). It is essential to use physiological approaches and mechanistic tools to understand how environmental stressors and alterations affect species at all levels of biological organization; organisms, populations and ecosystems (Wikelski and Cooke, 2006; Cooke *et al.*, 2013).

In the Estuary and Delta, severe declines in the abundance of several fishes have been documented over the past decades; referred to as the pelagic organism decline (Sommer *et al.*, 2007; Baxter *et al.*, 2008, 2010; Thomson *et al.*, 2010; Brooks *et al.*, 2012). Most affected are two introduced species, striped bass (*Morone saxatilis*) and threadfin shad (*Dorosoma pentenense*), and two native species, longfin smelt (*Spirinchus thaleichthys*) and delta smelt (*Hypomesus transpacificus*; Sommer *et al*., 2007; Thomson *et al*., 2010). The delta smelt is a pelagic fish species endemic to the Estuary and Delta and is listed as endangered and threatened under California State and Federal Endangered Species Acts, respectively (USFWS, 1993; CDFW, 2014). The observed decline in delta smelt abundance has led to the detection of genetic bottlenecks in years 2003, 2005 and 2007 (Fisch *et al.*, 2011). Delta smelt abundance has been associated with the low salinity zone (Moyle *et al.*, 1992) as well as with turbid water (Feyrer *et al.*, 2007; Grimaldo *et al.*, 2009; Bennett and Burau, 2015). The low salinity zone is centred around a salinity of 2 practical salinity units (PSU; Jassby *et al.*, 1995; Kimmerer *et al.*, 2013), with a variation in salinity of 0.5–6 PSU (Brown *et al.*, 2014), and is also characterized by the estuarine turbidity maximum (ETM; Peterson *et al.*, 1975; Schoellhamer, 2001). The ETM is described as an area of increased suspended particles, phytoplankton and zooplankton (Peterson *et al*., 1975; Roman *et al.*, 2001; Sanford *et al.*, 2001), thus representing a zone with higher turbidity and enhanced food sources, which is important for successful feeding and growth in many fish larvae (Dodson *et al.*, 1989; North and Houde, 2001). The turbidity in an ETM can be extremely variable and change quickly, e.g. shifts in turbidity levels from 20 to >150 NTU within 1 h have been observed in the ETM of the St Lawrence Estuary, Canada (Dodson *et al*., 1989).

Turbidity, often described as the cloudiness or murkiness of water, is defined as an expression of the optical property causing light scattering and light absorption rather than direct light transmission through a water sample (Rice *et al.*, 1994). Important factors contributing to the effect of turbidity are light intensity, suspended material and water depth (Lee and Rast, 1997), and these parameters interact with each other. In particular, the type of suspended material and its specific particle size, morphology, colour and refractive index influence the extent of light scattering and can affect vision and respiration at high densities (reviewed by Bruton, 1985). Along with other water physicochemical parameters, turbidity plays an important role in estuarine ecosystems. It is known, for example, to influence several trophic levels by altering species composition (Lunt and Smee, 2014) and can potentially affect the feeding strategy of fish (Hecht and Van der Lingen, 1992; De Robertis *et al.*, 2003; Johansen and Jones, 2013), as well as predator–prey interactions. Much research has been conducted on evaluating the effects of turbidity on the vision of fish and on predator–prey interactions (Gardner, 1981; Gregory and Northcote, 1993; Miner and Stein, 1996; Horppila *et al.*, 2004; reviewed by Utne-Palm, 2002). These studies assess the reaction distance between the fish and the prey, which is defined as the distance between the predator and prey at time of detection (Utne-Palm, 2002). Turbidity has been shown to affect fishes differentially, depending on their specific foraging type and associated behaviour. Large predatory fish, which detect prey visually from greater distances, are negatively affected by turbidity because the increased number of suspended particles can influence light scattering, thereby interfering with prey detection and potentially decreasing the reaction distance (Chesney, 1989; Giske *et al.*, 1994; Fiksen *et al.*, 2002; reviewed by Utne-Palm, 2002). Small planktivorous fish or larval fish, which detect prey from a shorter distance, may benefit from a degree of turbidity that increases their reaction distance (Miner and Stein, 1996; Utne-Palm, 2002). For instance, increased feeding in larval Pacific herring (*Clupea harengus pallasi*) was observed in mid-range turbidity levels compared with lower turbidity levels (Boehlert and Morgan, 1985). A certain turbidity level can have two advantages for small planktivorous and larval fish. First, turbidity can potentially enhance the contrast between the prey and its background (Hinshaw, 1985), allowing for easier feeding. Second, turbidity can lower the risk of predation from large predatory fish, thus providing a necessary level of safety (Gregory and Northcote, 1993), therefore influencing feeding and stress levels in fish. Furthermore, turbidity and suspended sediments have been shown to impact gills of fish, e.g. altered thickness of gill epithelium and significantly elevated numbers of mucous cells in clownfish larvae (*Amphiprion percula*; Hess *et al.*, 2015) as well as clogging of fish gill rakers and gill filaments as a result of excess suspended material (Sutherland and Meyer, 2007; Wong *et al.*, 2013). At large, negative effects of sediment particles of fish gills have been shown for several marine and freshwater fishes (Wilber and Clarke, 2001; Au *et al.*, 2004; Sutherland and Meyer, 2007; Wong *et al*., 2013; Hess *et al*., 2015).

In the mainstem Estuary and the Delta, turbidity levels vary greatly between locations, with values up to 220 Nephelometric Turbidity Units (NTU; Werner *et al.*, 2010) and occasional high peaks of 350 NTU (Bennett and Burau, 2015), e.g. during storm events. Turbidity in the Estuary and Delta has decreased over the past decades (Thomson *et al*., 2010), and several factors have been postulated to contribute to this decline, including the introduction of invasive species, such as clams, clearing the water (Carlton *et al.*, 1990; Nichols *et al.*, 1990; Thompson, 2005) and waterweed trapping sediment (Yarrow *et al.*, 2009). In addition, dam construction, sediment trapping in reservoirs and a reduction in major flood events have been discussed as causes of decreased turbidity (Jassby *et al.*, 2002; Wright and Schoellhamer, 2004; Jassby, 2008; Baxter *et al*., 2010). Reduced turbidity has also been associated with low primary productivity that results in a food-limited estuary (Kimmerer *et al.*, 2005).

Turbidity is crucial to the completion of the delta smelt's life cycle. Delta smelt have been reported to use turbid waters to hide from predators (Moyle, 2002), and it has been hypothesized that increased pulse turbidity from first flush events (first winter storm event of the year) might be a cue for the annual spawning migration of delta smelt to the northern Delta (Grimaldo *et al*., 2009; Sommer *et al.*, 2011). Furthermore, laboratory studies on the feeding response of early life stages of delta smelt found a positive relationship between turbidity and feeding behaviour (Baskerville-Bridges *et al.*, 2004, 2005), and for juveniles (120 days post-hatch; dph) constant feeding up to 120 NTU with a significant decrease in food intake at 250 NTU was observed (Hasenbein *et al.*, 2013). However, there is still a lack of information on turbidity tolerance ranges for delta smelt, because postulated associations, as they relate to this species of concern, have not been fully evaluated. There is a great need to identify tolerance ranges of delta smelt for turbidity in order to manage water diversions and water pumping more efficiently. Knowing tolerance ranges for this sensitive species will help managers make informed decisions about delta smelt conservation. Moreover, knowledge of the turbidity preference is also crucial for conservation programmes that aim at captive breeding of this species, as well as for maintaining optimal holding conditions in ecological and ecotoxicological studies involving this species.

The aim of the present study was to evaluate short-term effects of environmentally relevant turbidities (5–250 NTU) on the physiological stress responses of late-larval delta smelt, with the goal of determining preferred turbidity levels and tolerance ranges under well-defined conditions. We focused on the larval life stage because it has been previously shown to be the most sensitive life stage (Connon *et al.*, 2009, 2011a,b; Komoroske *et al.*, 2014), dependent on turbidity for feeding (Baskerville-Bridges *et al*., 2004; Lindberg *et al.*, 2013) and other aspects of life history (Lindberg *et al*., 2013), and tolerance to turbidity has not been tested. We hypothesized that different turbidity levels would affect overall physiological stress, which would in turn affect survival, but that maximal prey capture ability (feeding) would occur at specific turbidity requirements. Thus, in order to determine the preferred range of turbidities for late-larval delta smelt, we conducted integrative assessments using a combination of physiological endpoints (gene transcription, plasma hormones and metabolites) and contrasted these with ecological performance measures (food intake and survival).

## Materials and methods

### Study animals

Late-larval delta smelt (*H. transpacificus*; 60 dph) were provided by the Fish Conservation and Culture Laboratory (FCCL) UC Davis in Byron, CA, USA, where feeding ability and physiological response tests were conducted. Tests were conducted during 12–16 August 2013. The start and end of the test were mornings at 9.00 h. Fish were cultured according to culture protocols described by Baskerville-Bridges *et al.* (2005) and Lindberg *et al.* (2013). In brief, fish were cultured in black tanks without substrate, plants or structure, because this resembles their natural habitat in the open pelagic zone. A recirculating system connected to biofilters was used to maintain larvae with a feeding regimen of six feedings per day. Larvae were fed with newly hatched *Artemia franciscana* at a volume in the tank of 1–3 nauplii ml^−1^. The average rearing temperature throughout the rearing period was 17.4°C (±0.05 SE). The mean length (fork length) and weight of 60 dph delta smelt were 19.37 mm (±0.09 SE) and 0.0303 g (±0.00 SE), respectively. Fish were held at turbidities of 9.94 NTU (±0.25 SE) for a period of 8 weeks before the test. The light intensities for larval and late-larval life stages are kept at low levels of 4–5 and 1–2 µmol m^2−1^ s^−1^, respectively (Lindberg *et al*., 2013). The low light level accommodates for the increased light sensitivity of this fish species (Lindberg *et al*., 2013).

### Fish exposures

Studies were set up to evaluate feeding success and physiological stress responses to varying turbidities. Late-larval delta smelt (60 dph) were exposed, at a stocking density of 30 fish per vessel, to turbidities of 5, 12, 25, 35, 50, 80, 120 and 250 NTU (nominal values) in aerated facility water in 8 l black circular fish tanks (static water system; 2 Gallon Black Plastic Pail; Item # 3539; United States Plastic Corporation^®^, USA), for a period of 24 h with a light–dark cycle of 16 h–8 h. Black exposure vessels were used because they are considered a necessary component for successful rearing and culture (Lindberg *et al*., 2013). Fish stocking density was determined in earlier studies (Hasenbein *et al.*, 2016) to be in the optimal range of four to eight fish per litre. Salinity was kept constant at 2 PSU for the feeding test, whereas the physiology test had a salinity of 0.2 PSU. *Nannochloropsis* algae (Nanno 3600 – High yield grow out feed; Reed Mariculture Inc., USA) were spiked into exposure vessels to achieve desired turbidities. Light was provided by a Lithonia Lighting Fluorescent Luminaire (Bulb: Philips F40T12/DX, 40 W), and light intensity was kept constant at a low level of 48 lx (±1.13 SE). The exposure vessel and light intensity were chosen according to culture protocols (Lindberg *et al*., 2013). Fish tanks were aerated using air stones with a constantly low aeration rate to keep algae suspended. Aeration had to be limited because culture methods recommend a gentle aeration to account for early life-stage sensitivity (Baskerville-Bridges *et al*., 2005). Fish were fed *Artemia* in the facility tanks prior to test set-up, but were kept unfed throughout the duration of the test.

For both tests, physicochemical water parameters were monitored at test initiation and at termination. Dissolved oxygen was measured using a YSI Model 55 DO meter (YSI Inc. Xylem Inc., Yellow Springs, OH, USA). Salinity and specific conductance were measured using a YSI Model 63 Multimeter (YSI Inc. Xylem Inc., Yellow Springs, OH, USA). pH was measured using a waterproof portable pH meter (Hanna Instruments Model HI9124; Hanna Instruments Inc., Woonsocket, RI, USA). Turbidity was measured with a Hach 2100q portable turbidity meter (Hach Company, Loveland, CO, USA) that conducts a ratio turbidimetric determination using a primary nephemoletric light scatter signal (90° angle) to the transmitted light scatter signal. A water sample was taken from the exposure vessel, shaken for 10 s and placed into the turbidity meter. Ammonia was measured using a Hach pocket colorimeter II Filter Photometer (Hach Company). Ammonia was determined in control treatments only, because the color of the *Nannochloropsis* algae interfered with the measurement method. Temperature was measured at 1 min intervals using iBCod submersible temperature loggers (Alpha Mach Inc., Ste-Julie, QC, Canada). Light levels were measured directly above the water surface of the exposure vessel using an Extech instruments easy view 30 light meter (FLIR commercial system Inc., Nashua, NH, USA).

#### Feeding test

After 24 h, subsets of fish were fed with live newly hatched *Artemia fransciscana* in solution [100 ml per bucket; density of 462 *Artemia* per ml (±21 SE); total food density in the vessel was five to six *Artemia* per millilitre of water] for a period of 7.5 min (duration determined in preliminary tests to result in 50% gut fullness; M. Hasenbein, R. E. Connon, N. A. Fangue and J. Geist, unpublished data). *Artemia franciscana* was chosen as a food item because this is the same as used in the culture procedures. At test termination, dead fish were identified, counted and discarded. Surviving fish were immediately euthanized with an overdose of tricaine methanesulfonate (MS-222; Finquel, USA) buffered with sodium bicarbonate. Specimens were transferred into 15 ml tubes filled with 70% ethanol and preserved until further processing for stomach content analysis. Fish were measured for weight and length and dissected for gut content (number of *Artemia* ingested) under a microscope (×40 resolution). Survival was recorded at test termination and the percentage of survival was calculated based on fish numbers at test set-up and test termination. Tests were conducted in quadruplicate, except for the 120 NTU treatment, which had only three replicates owing to vigorous aeration in one of the replicates resulting in elevated mortality.

#### Physiology test

At test termination, subsets of unfed fish were immediately euthanized as described in the previous subsection. Specimens were transferred into 1.5 ml tubes (Eppendorf) and subsequently snap frozen in liquid nitrogen. Samples were stored at −80°C for subsequent biochemical and molecular analyses.

### Cortisol assessments

Methods for cortisol measurements are described by Hasenbein *et al*. (2016). In brief, whole-body cortisol was assessed in a total of four to seven fish from each treatment depending on survival, using modified methods established for zebrafish (Alsop and Vijayan, 2008; Cachat *et al.*, 2010). Volumes of solutions were optimized for use in juvenile delta smelt (Hasenbein *et al*., 2013) and further enhanced for larval fish. Samples were defrosted on ice and homogenized in 1× phosphate-buffered saline (PBS) using a TissueLyzer LT. The resulting homogenate was divided into equal amounts of 500 µl and used for cortisol and total protein determination. Cortisol was eluted from the homogenate using diethyl ether. Ether was then allowed to evaporate, and dried cortisol samples were resuspended in 1× PBS. Cortisol assessments were performed according to manufacturer's instructions (Salivary Cortisol, Enzyme Immunoassay Kit; Salimetrics, Inc., State College, PA, USA), and levels (in milligrams per decilitre) were calculated with a four-parameter sigmoid standard curve (minus curve fit). Cortisol levels were normalized to total protein and denoted as cortisol concentration (picograms of cortisol per microgram of protein). The second half of the homogenate was used to determine protein content. After centrifugation at 16 500***g*** for 30 min at 4°C, the supernatant of each sample was collected and used for total protein content determination following the manufacturer's protocol (BCA Protein Assay Kit; Thermo Fisher Scientific Inc., Waltham, MA, USA).

### Quantitative polymerase chain reaction

Total RNA was extracted from whole-body homogenates. RNA extractions were performed according to the manufacturer's protocols using the RNeasy Mini Qiacube Kit (Qiagen^©^, Venlo, Limburg, The Netherlands). Qualitative and quantitative RNA determination was conducted using a NanoDrop ND1000 Spectrophotometer; 260/280 and 260/230 ratios ranged from 2.04 to 2.18 and from 1.94 to 2.35, respectively. Integrity of total RNA was assessed by electrophoresis on a 1% (w/v) agarose gel. Depending on survival, the stress response and physiology gene transcription of five to eight fish per treatment were assessed by quantitative polymerase chain reaction (qPCR). Complementary DNA (cDNA) synthesis was performed using Reverse Transcriptase III (SuperScript^®^ III Reverse Transcriptase; Invitrogen^TM^, Life technologies^TM^, Carlsbad, CA, USA), and primers and probes for qPCR analyses were designed using Roche Universal Library Assay Design Center (https://www.roche-applied-science.com). Quantitation of transcription was performed using SDS 2.4 software (Applied Biosystems^®^, Life technologies^TM^). Responding genes were normalized using a normalization factor calculated based on the geometric mean of two control genes, namely *glyceraldehyde-3-phosphate dehydrogenase* (*gapdh*) and *beta-actin* (*b-actin*). Normalization was performed according to the ‘geNorm’ algorithm version 3.5 as described by Vandesompele *et al.* (2002).

A total of 17 target genes were selected based on their involvement in the hypothalamic–pituitary–interrenal axis and in other important functions, such as energy metabolism, development and somatic growth, ion homeostasis, oxygen homeostasis, inflammatory response, osmoregulation and general stress. Selected genes (gene name and code), function, primer sequence and efficiency are listed in Table 1.

**Table 1:** Primer and probe sequences of genes used as molecular biomarkers to determine stress levels in late-larval delta smelt (*Hypomesus transpacificus*)

Gene name	Gene code	Primer sequences	Function	Probe no.	Percentage efficiency
*Glutathione-*S*-transferase*	*gst*	5′ → 3′ AATCTCCCTGGCAGACATTGTT 3′ → 5′ GGCCGGCTCTCAAACACAT	Cellular detoxification, osmotic stress	127	108
*Mineralocorticoid receptor 1*	*mr1*	5′ → 3′ TTTCTACACTTTCCGCGAGTCA 3′ → 5′ TGATGATCTCCACCAGCATCTC	Mediator for cortisol signalling; HPI axis	39	99
*Glucocorticoid receptor 2*	*gr2*	5′ → 3′ CATCGTGAAGCGTGAGGAGAA 3′ → 5′ TGCATGGAGTCCAGTAGTTTGG	Mediator for cortisol signalling; HPI axis	129	98
*Pro-opiomelanocortin*	*pomc*	5′ → 3′ TGTTCACCTGTGCAGGTCTGA 3′ → 5′ GAGAAGCTCTCTTCCGTGGACA	Precursor of adrenocorticotrophic hormone; HPI axis	127	102
*11-beta-hydroxyteroid-dehydrogenase type 1*	*11-beta-hsd-1*	5′ → 3′ CGTGTCGTCTCTGCTGGCTA 3′ → 5′ GGCGAACTTGGTGGAGGAG	Cortisol conversion; HPI axis	55	109
*11-beta-hydroxyteroid-dehydrogenase type 2*	*11-beta-hsd-2*	5′ → 3′ TCCTGCCATCCTCCTACAAGAC 3′ → 5′ TCTGGACCAGGTGTTTGAACTG	Cortisol conversion; HPI axis	14	106
*Beta actin*	*β-actin*	5′ → 3′ TGCCACAGGACTCCATACC 3′ → 5′ CATCGGCAACGAGAGGTT	Housekeeping gene, reference gene	11	107
*Glyceraldehyde 3-phosphate dehydrogenase*	*gapdh*	5′ → 3′ TCCACGAGAAAGACCCAACT 3′ → 5′ CACGCCAGTAGACTCAACCA	Housekeeping gene, reference gene	159	95
*Insulin like growth factor*	*igf*	5′ → 3′ GACACGCTGCAGTTTGTATGTG 3′ → 5′ CCATAGCCTGCCGGTTTGT	Development, somatic growth, interaction with growth hormone	110	100
*Serum/glucocorticoid regulated kinase*	*sgk3*	5′ → 3′ TTATTGAGATCAAAGCGCATGAC 3′ → 5′ GGTGTGAAGGGAGGTGGAATC	Ion homeostasis, stimulates sodium transport via epithelial sodium channel	85	107
*Hypoxia inducible factor 1 alpha*	*hif1a*	5′ → 3′ GCCATGGCCAAGCTCCTTA 3′ → 5′ ATTCAGCATTGGCACTAAGCAC	Transcription factor, oxygen homeostasis	41	105
*Glucose transporter 2*	*glut2*	5′ → 3′ GCCATGTCAGTTGGCCTCAT 3′ → 5′ GACATGCTGACGTAGCTCATCC	Glucose homeostasis, energy metabolism	130	109
*Heat shock protein 70 kD*	*hsp70*	5′ → 3′ AAGATTCTGGAGAAGTGCAACGA 3′ → 5′ CCTTCTCAGCGGTCTGGTTCT	General stress, heat stress	20	107
*Nuclear factor k-beta*	*nfkb*	5′ → 3′ TGCACGGATGAACACATTGTC 3′ → 5′ CCAAAGTCCAGAGGCTTGTCA	Transcription factor, host defense, chronic inflammatory diseases	123	103
*Ammonium transporter*	*NH4 trans*	5′ → 3′ CAGGCTGTCTTATCGCTTACGG 3′ → 5′ CAGCGTCATGACTAACAGCTGAA	Excesss ammonia elimination across gills and skin	61	95
*Catalase*	*catalase*	5′ → 3′ GACCAGGGCATCAAGAACCTTA 3′ → 5′ GGATGGCGTAGTCTGGGTCA	Antioxidant enzyme, protects cell from oxidative damage by reactive oxygen species	88	97
*Sodium potassium ATPase*	*Na K atpase*	5′ → 3′ GTCATCCCAATCTACTGCACCA 3′ → 5′ CATGATGTCGCCAATCTTGC	Ion transport during osmoregulation, osmoregulatory stress	88	109

Abbreviations: HPI axis, hypothalamic–pituitary–interrenal axis.

### Statistical analysis

Data were analysed using the ‘*stats*’ package in R-project for statistical computing (version 3.0.2; http://www.r-project.org; R-CoreTeam, 2014). All data sets were tested for normal distribution and homoscedasticity using the Shapiro–Wilk normality test and the Fligner–Killeen test. Cortisol data were logarithmically transformed to meet normality criteria. Data were tested for effects of turbidity using a one-way ANOVA if normally distributed or a Kruskal–Wallis test if non-normally distributed. Turbidity was defined as the predictor (categorical variable) and the respective response variable (feeding, mortality, cortisol or qPCR) as the continuous variable. Where turbidity showed a significant effect, data were analysed for *post hoc* contrasts between treatments using Tukey's HSD *post hoc* test or the multiple comparison test after Kruskal–Wallis test. Statistical decisions were based on an α level of 0.05. Further statistical information is presented in supplementary Table S1.

Principal component analysis (PCA) was carried out on the transcriptomic (qPCR) data set in order to analyse differences in transcription patterns between treatments and to determine to what extent these profiles were affected by the treatments. The PCA scores were calculated using the covariance matrix, and principal components 1, 2 and 3 (PC1, PC2 and PC3) were determined to explain the majority of the variation in the data, using a Scree test as described by D'Agostino and Russell (2005). PCA scores of PC1, PC2 and PC3 were plotted as a centroid graph to describe, interpret, visualize and support clustering of treatments with similar transcriptomic responses. In addition, the respective biplot was plotted to identify genes driving the two components and explain the percentage variation.

## Results

### Physicochemical parameters

Physicochemical water parameters for the feeding test and the physiology test are presented in Tables 2 and 3, respectively. Specific conductance, pH, salinity and temperature remained stable throughout the test duration. Dissolved oxygen declined slightly over time in all turbidity levels in the feeding test and in all but one turbidity level of the physiology test. Values ranged over time for the feeding test from 9.79 to 7.44 mg l^−1^ and for the physiology test from 9.53 to 7.75 mg l^−1^, respectively. At all turbidity levels of the feeding test and at turbidity levels 25, 35, 50, 80, 120 and 250 NTU of the physiology test, a decrease in turbidity was observed at the 24 h time point because delta smelt require only gentle aeration (Table 3). Ammonia concentration increased over time from 0.08 mg l^−1^ (±0.01 SE) at test initiation to 0.15 mg l^−1^ (±0.00 SE) at test termination.

**Table 2:** Physicochemical water parameters of the turbidity feeding test conducted on late-larval delta smelt 60 days post-hatch exposed to different levels of turbidity over 24 h

Treatment turbidity (NTU) nominal concentration	Average SE	DO (mg l^−1^)		Spec. Con. (µS cm^−1^)		pH		Salinity (PSU)		Turbidity (NTU) measured concentration		Ammonia (mg l^−1^) NH_3_-N		Light intensity (lx)	Temperature (°C)
		T0	T24	T0	T24	T0	T24	T0	T24	T0	T24	T0	T24		
5 NTU	Average	9.79	8.84	3738	3804	7.65	7.22	2.00	2.03	6.18	5.16	Average = '0.08 ± 0.01 SE	Average = '0.15 ± 0.00 SE	Average = '48.07 ± 1.13 SE	Average = '17.52 ± 0.00 SE
	SE	0.05	0.05	82	102	0.07	0.04	0.00	0.05	0.22	0.25				
12 NTU	Average	9.48	8.88	3712	3852	7.82	7.32	2.05	2.05	11.70	10.6				
	SE	0.05	0.08	238	150	0.06	0.02	0.09	0.09	0.20	0.20				
25 NTU	Average	9.55	8.64	3721	3732	7.89	7.48	1.98	1.98	24.20	19.90				
	SE	0.03	0.15	129	141	0.03	0.04	0.08	0.08	1.00	0.80				
35 NTU	Average	9.54	8.59	3941	3939	7.88	7.47	2.10	2.10	34.80	27.30				
	SE	0.06	0.11	69	72	0.02	0.02	0.04	0.04	0.80	0.70				
50 NTU	Average	9.45	8.42	3699	3743	7.87	7.47	1.93	2.00	51.30	43.80				
	SE	0.15	0.11	24	45	0.04	0.01	0.03	0.00	1.00	3.10				
80 NTU	Average	9.51	8.35	3932	3887	7.93	7.53	2.05	2.05	80.70	58.20				
	SE	0.10	0.24	65	118	0.07	0.03	0.06	0.06	1.40	5.60				
120 NTU	Average	9.57	8.44	3887	3845	7.90	7.52	2.07	2.07	120	97.0				
	SE	0.04	0.14	129	174	0.01	0.05	0.07	0.09	2	5				
250 NTU	Average	9.24	7.44	3760	3909	8.03	7.55	2.05	2.08	251	199				
	SE	0.08	0.21	188	62	0.08	0.03	0.06	0.05	2	4				

Values are means with SE across replicates for each turbidity level taken at test initiation T0 and test termination T24. Abbreviations: DO, dissolved oxygen; NTU, nephelometric turbidity unit; PSU, practical salinity unit; Spec. Con., specific conductance; T0, time point at test start; T24, time point at test termination.

**Table 3:** Physicochemical water parameters of the turbidity physiology test conducted on late-larval delta smelt 60 days post-hatch exposed to different levels of turbidity over 24 h

Turbidity (NTU) nominal concentration	DO (mg l^−1^)		Spec. Con. (µS cm^−1^)		pH		Salinity (PSU)		Turbidity (NTU) measured concentration		Ammonia (mg l^−1^) NH_3_-N		Light intensity (lx)	Temperature (°C)
	T0	T24	T0	T24	T0	T24	T0	T24	T0	T24	T0	T24		
5	9.47	9.29	764	864	8.17	8.23	0.4	0.4	6.38	6.30	Average = '0.08 ± '0.01 SE	Average = '0.15 ± 0.00 SE	Average = '48.07 ± '1.13 SE	Average = '17.52 ± 0.00 SE
12	9.53	9.45	692	890	8.63	8.09	0.3	0.4	8.8	11.8				
25	9.43	9.15	768	1083	8.36	8.02	0.4	0.4	26.4	21.9				
35	9.28	9.09	882	858	8.54	7.8	0.5	0.4	32.2	27.3				
50	8.41	9.19	853	864	8.56	7.74	0.4	0.4	53.2	42.5				
80	9.34	7.75	887	1131	8.49	7.69	0.4	0.5	82.2	62.5				
120	9.32	8.62	1017	1082	8.50	7.76	0.5	0.5	129	123				
250	9.29	8.62	910	1163	8.78	8.41	0.4	0.6	240	202				

Values for each exposure vessel and respective turbidity level were obtained at test initiation T0 and test termination T24.Abbreviations: DO, dissolved oxygen; NTU, nephelometric turbidity units; PSU, practical salinity unit; Spec. Con., specific conductance; T0, time point at test start; T24, time point at test termination.

### Feeding test

#### Survival

**Figure 1:**
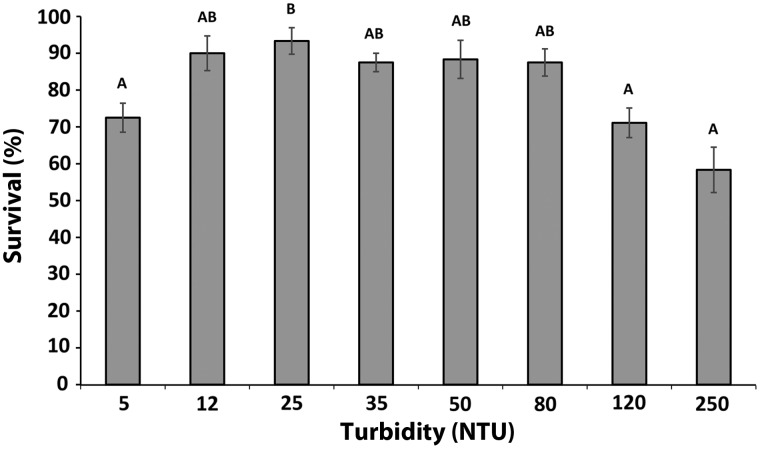
Percentage survival of 60 days post-hatch late-larval delta smelt after 24 h exposure at turbidity levels between 5 and 250 NTU (*n* = 3–4). Values are calculated using the average across replicates (exposure vessels). Bars depict standard error (SE). Different letters indicate significant differences between turbidity levels (ANOVA, Tukey's HSD test, α = 0.05).

Mean percentage survival was 81% (±4.34 SE) across all treatments (Fig. 1). Highest survival rates were observed in treatments 12, 25, 35, 50 and 80 NTU, with survival rates of 90 (±4.71 SE), 93 (±3.60 SE), 87 (±2.50 SE), 88 (±5.18 SE) and 88% (±3.69 SE), respectively. Survival decreased at low turbidities of 5 NTU, averaging 73% (±3.94 SE), as well as at high turbidities of 120 and 250 NTU, averaging 71 (±4.01 SE) and 58% (±6.16 SE), respectively.

#### Feeding

**Figure 2:**
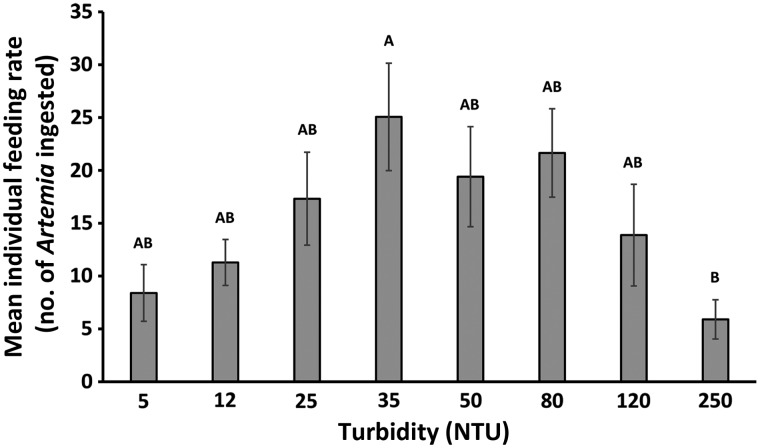
Mean number of *Artemia franciscana* ingested by individual 60 days post-hatch late-larval delta smelt following 24 h exposure at turbidity levels between 5 and 250 NTU (*n* = 3–4). Values are calculated using the average across replicates (exposure vessels). Bars depict standard error (SE). Different letters indicate significant differences between turbidity levels (ANOVA, Tukey's HSD test, α = 0.05).

Highest feeding rates (number of *Artemia* ingested) were recorded at mid-range turbidities of 25, 35, 50 and 80 NTU (Fig. 2), where late-larval delta smelt ingested a mean of 17 (±4.40 SE) *Artemia* per fish at 25 NTU, 25 (±5.08 SE) at 35 NTU, 19 (±4.75 SE) at 50 NTU and 22 (±4.19 SE) at 80 NTU. Lower feeding rates were observed at both low and high turbidities, with prey ingestions of 8 (±2.68 SE) and 11 (±2.17 SE) *Artemia* per fish at turbidities of 5 and 12 NTU, respectively, and 14 (±4.81 SE) and 5 (±2.07 SE) *Artemia* per fish at turbidities of 120 and 250 NTU, respectively. Significant differences between feeding rates were detected between 35 and 250 NTU (*P* < 0.05). No ingested algae were found in fish guts.

### Physiology test

#### Cortisol

**Figure 3:**
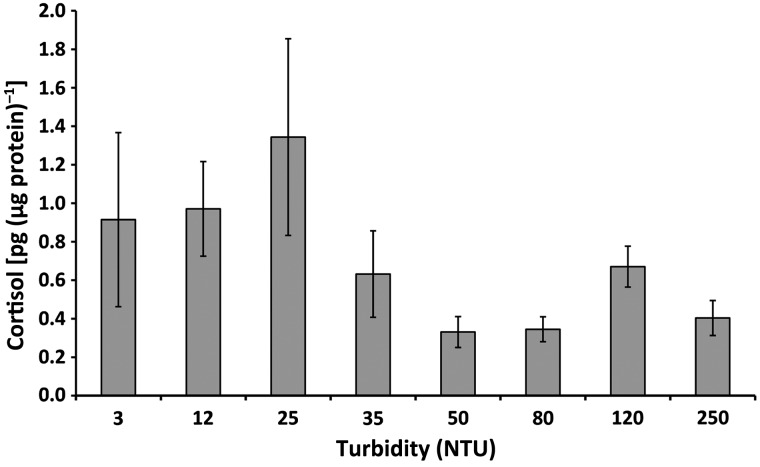
Whole-body cortisol levels of 60 days post-hatch late-larval delta smelt following 24 h exposure at turbidity levels between 5 and 250 NTU. Values are calculated using the average across individual fish (*n* = 4–7). Bars depict standard errors (SE).

No statistically significant differences in whole-body cortisol levels were observed in delta smelt maintained at turbidities ranging from 5 to 250 NTU (Fig. 3). However, cortisol values tended to be lowest in fish maintained at 50 NTU and above, whereas they were elevated and more variable in fish held at the lower turbidity levels of 5, 12 and 25 NTU.

#### Quantitative PCR

Changes in gene transcription for all 15 genes measured with qPCR are presented in Table 4 and in Supplementary Table S2. Four out of 15 genes, namely *gst*, *hsp70*, *glut2* and *NH4 trans*, responded significantly in delta smelt (Table 4). *gst* was significantly differentially expressed at the highest turbidity (250 NTU), relative to 25 (*P* < 0.05) and 35 NTU (*P* < 0.05), suggesting oxidative stress at higher turbidity. *hsp70* indicated highest general stress levels at 120 and 250 NTU, significantly different from the 5 (*P* < 0.01), 12 (*P* < 0.001) and 35 NTU (*P* < 0.05) groups. No significant differences were observed at 25 NTU, owing to high variability in responses. *glut2* was also highest at high turbidity, significantly differentiating between 250 and 5 NTU (*P* < 0.01), 35 (*P* < 0.05) and 80 NTU (*P* < 0.05). Interestingly, *NH4 trans*, a gene coding for the ammonia transporter, also responded significantly in fish maintained at 12 NTU relative to 120 (*P* < 0.001) and 250 NTU (*P* < 0.05).

**Table 4:** Fold-change in gene transcription for statistical significant genes measured in late-larval delta smelt 60 days post-hatch maintained for 24 h at different turbidity levels

Turbidity	*hsp70*	*glut2*	*NH4 trans*	*gst*
5				
Average	2.10^a^	2.32^a^	1.28^ab^	2.16^ab^
SE	0.28	0.24	0.22	0.31
12				
Average	1.83^a^	2.99^ab^	0.71^a^	2.25^ab^
SE	0.25	0.37	0.16	0.27
25				
Average	2.26^ab^	2.80^ab^	1.60^ab^	1.87^a^
SE	0.39	0.55	0.42	0.36
35				
Average	2.76^a^	2.79^a^	1.85^ab^	2.00^a^
SE	0.41	0.38	0.43	0.21
50				
Average	3.48^ab^	3.90^ab^	1.90^ab^	3.00^ab^
SE	0.28	0.54	0.22	0.39
80				
Average	3.22^ab^	3.43^a^	1.40^ab^	2.87^ab^
SE	0.23	0.56	0.22	0.51
120				
Average	4.27^b^	4.82^ab^	3.05^b^	3.92^ab^
SE	0.45	0.6	0.62	0.71
250				
Average	3.62^b^	6.97^b^	2.13^b^	5.36^b^
SE	0.6	1.68	0.59	1.68

Values above 1.0 indicate upregulated genes and values below 1.0 indicate downregulated genes. Statistically significant differences between turbidity levels as detected by one-way ANOVA and Tukey's HSD *post hoc* test (significance level α = 0.05) are indicated by superscript letters a and b. Abbreviations: *glut2*, *glucose transporter 2*; *gst*, *glutathione*-S-*transferase*; *hsp70*, *heat shock protein 70 kD*; *NH4 trans*, *ammonium transporter*.

#### Principal component analysis

**Figure 4:**
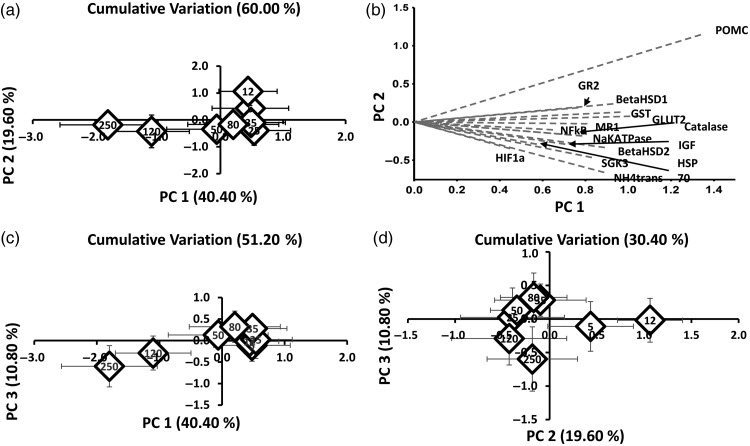
Graphical depiction of the scores of principal components (PC) 1 and 2 (**a**); 1 and 3 (**c**); 2 and 3 (**d**); centroid graph for each treatment. Percentages indicate the amount of variation explained by the respective PC. Numbers indicate turbidity levels in nephelometric turbidity units (NTU). (**b**) Respective biplot indicates the genes driving the clustering of PC1 and PC2, with gene names. Abbreviations: *11-beta-hsd-1*, *11-β-hydroxysteroid-dehydrogenase-type 1*; *11-beta-hsd-2*, *11-β-hydroxysteroid-dehydrogenase-type 2*; *glut2*, *glucose transporter 2*; *gr2*, *glucocorticoid receptor 2*; *gst*, *glutathione*-S-*transferase*; *hif1a*, *hypoxia inducible factor 1 alpha*; *hsp70*, *heat shock protein 70 kD*; *igf*, *insulin like growth factor*; *Na K atpase*, *sodium potassium atpase*; *nf-kb*, *nuclear factor k-beta*; *mr1*, *mineralocorticoid receptor 1*; *NH4 trans*, *ammonium transporter*; *pomc*, *pro-opiomelanocortin*; *sgk3*, *serum/glucocorticoid regulated kinase 3*.

Principal component analysis was conducted on all gene transcription data (Table 4 and Fig. 4). Principal component 1 explained 40.40% of the variation, whereas PC2 and PC3 explained 19.60 and 10.80% of the variation, respectively, leading to a cumulative variation of 70.80% (Fig. 4). Plotting PC1, PC2 and PC3 against each other for visual evaluation revealed trends in differentiation and clustering of treatments with similar transcriptomic responses. The plot for PC1 vs. PC2 (Fig. 4a) clustered treatments 25, 35, 50 and 80 NTU more closely with lower turbidity treatments (5 and 12 NTU) than with higher turbidity treatments (120 and 250 NTU). The respective biplot (Fig. 4b) indicates that several different genes, such as *gr2*, *mr1*, *11-beta-hsd-1*, *glut2*, *gst* and *catalase*, loaded heavily on PC1, whereas *pomc*, *hif1a*, *sgk3*, *hsp70* and *NH4 trans* determined the loading for PC2. Differentiation of the highest turbidities (120 and 250 NTU) from all others tested was more pronounced when plotting PC1 vs. PC3 (Fig. 4c). In contrast, the plot for PC2 vs. PC3 (Fig. 4d) highlighted a more pronounced differentiation of the lowest turbidity treatments from mid-range and high turbidity treatments.

## Discussion

In this study, the effects of different levels of turbidity on late-larval delta smelt survival, feeding and physiological stress responses were quantified over a period of 24 h. Overall, turbidities from 25 to 80 NTU were determined to be the optimal range in the tested conditions as evident from the highest survival, feeding and changes in gene expression compared with other treatments. Detrimental effects occurred both at low turbidities of 5 and 12 NTU and at high turbidities of 120 and 250 NTU. Consequently, even short-term (<24 h) exposure to such conditions is likely to have significant adverse effects on delta smelt.

How turbidity affects an organism is influenced by light intensity, water depth and the type of suspended material (Lee and Rast, 1997); these factors can vary in the field depending on local habitat conditions, but can be controlled in laboratory assessments. Standardized turbidity loading experiments in the laboratory are conducted using several different types of particles, e.g. soil (Mussen *et al.*, 2012), bentonite (Vogel and Beauchamp, 1999; Quesenberry *et al.*, 2007), red clay (Swenson and Matson, 1976), clay (Ardjosoediro and Ramnarine, 2002), natural sediment (Sirois and Dodson, 2000) and planktonic algae (Ajemian *et al.*, 2015). Although these experiments are not perfect analogues of field conditions, our data correspond well with field observations where delta smelt are associated with specific water turbidity levels (10–50 NTU; Feyrer *et al*., 2007; Grimaldo *et al*., 2009; Bennett and Burau, 2015).

Delta smelt survival was highest at mid-range turbidities, but reduced at very low and high turbidities. The reduced survival at low turbidities may be explained by elevated light intensity resulting in increased stress levels (supported by trends in cortisol levels). Smelts in general have been reported to be extremely light sensitive (Dembiński, 1971; Heist and Swenson, 1983; Appenzeller and Leggett, 1995; Horppila *et al*., 2004), an attribute also reported for delta smelt (Lindberg *et al*., 2013). Smelts in the wild have been observed actively avoiding waters with high light intensities (Dembiński, 1971; Heist and Swenson, 1983; Appenzeller and Leggett, 1995). Restricted water depth in the exposure vessel prevents the fish from escaping deeper into the water column in order to avoid elevated light intensities, which is possible in natural environments. Turbidity and light intensity are therefore likely to be key factors determining their location in the water column. Another potential cause for the elevated stress and reduced survival observed at low turbidity levels could stem from the concept that elevated turbidity is beneficial for planktivorous fish, in order to hide from predators. In fact, delta smelt are postulated to use turbid waters to hide from predators (Moyle, 2002). The ‘turbidity as a cover’ hypothesis as described by Lehtiniemi *et al.* (2005, and references therein) might play an important role in explaining the results of the present study. Although turbid conditions might minimize the perceived risk of predation, some predators might be able to take advantage of reduced anti-predator behaviours in these conditions.

Reduced survival at high turbidities could be caused by altered gill function, reducing the fish's capacity for respiration (Mallatt, 1985; Evans *et al.*, 2005; Hess *et al*., 2015). Clogging of fish gill rakers and gill filaments as a result of excess suspended material is not uncommon (Bruton, 1985; Sutherland and Meyer, 2007; Wong *et al*., 2013). Although not determined in the present study, it is possible that algal material restricted respiration by filling the fish's gill cavities or affecting gill morphology, influencing function. The increased expression of *gst* at high turbidity in delta smelt in this test is an indication of oxidative stress, which could also result in osmotic imbalance, as well as overall effects on respiration. Highest survival rate across mid-range turbidities (12–80 NTU) indicates the optimal range for late-larval delta smelt in test conditions. However, increased stress levels and reduced feeding were determined in treatment 12 NTU, where mortality was low, indicating sub-lethal stress levels in fish exposed to this treatment in test conditions.

Feeding responses corresponded to survival as expected. A constantly high feeding rate was observed between 25 and 80 NTU, with a peak at 35 NTU. A similar result was found in studies on European smelt that tested the feeding response to turbidities and light intensities; determining highest feeding rates at 20 NTU and a constant feeding between 20 and 50 NTU (Horppila *et al*., 2004). This feeding pattern is typical for planktivorous fish, which benefit from turbid waters by a contrast enhancement that helps them to detect prey items (Boehlert and Morgan, 1985; Utne-Palm, 2002). Studies on larvae of Pacific herring (*Clupea harengus pallasi*) that assessed the feeding response to different concentrations of suspended sediments, found increased feeding rates at intermediate concentrations compared with reduced feeding rates in control treatments (no suspended sediments) and at very high concentrations (Boehlert and Morgan, 1985). Increased stress can lead to reduced food intake and food conversion rate in fish (Wendelaar Bonga, 2011), thus the observed low feeding rates at low turbidity levels may be related to increased stress levels, indicated by trends in cortisol. However, low feeding rates could also be attributed to backscattering of light. Light intensities above the light saturation level of the fish's physiological capacities (sensitivity and vision) cause negative effects on prey detection (Utne-Palm, 2002) by minimizing the contrast between the background and the prey item, thus making it less visible (Cerri, 1983; Guthrie, 1986; Loew and McFarland, 1990). This would reduce the ability of the delta smelt to find prey, leading to decreased feeding rates. At high turbidities, reduced feeding is also likely owing to elevated stress levels, and this is consistent with assessed molecular indices of oxidative and osmotic stress. As with backscattering of light at low turbidity, reduction in feeding at high turbidity may also be caused by an impaired field of vision. Even though larval and juvenile delta smelt are planktivorous feeders, it is highly likely that they cope with a wide range of turbidities; however, if turbidities reach levels where visibility is impaired, prey detection may become impossible. We demonstrated reduced feeding at high turbidities (250 NTU) in prior studies on juvenile delta smelt (Hasenbein *et al*., 2013). Furthermore, high turbidities can reduce the reactive distance of a fish (Hecht and Van der Lingen, 1992; Miner and Stein, 1996; Utne, 1997; Utne-Palm, 2002) and with that, limit the volume of water that a fish could search for prey. The observed feeding in 120 and 250 NTU would therefore be resultant of incidental prey encounters at close proximities. Feeding assessments were conducted using *A. franciscana*, which is used in the culture procedures, and fish were fed in abundance. It is likely that feeding results presented here would be qualitatively similar if natural prey items were used, but absolute feeding performance values may differ markedly as a result of factors such as differential prey mobility and escape responses. Larval fish are constantly swimming and do not perform a coordinated hunt, rather feeding when directly encountering prey items (Bennett, 2005). In future studies, feeding performance could be explored further by using numerous prey species, including numerous zooplankton species that are commonly consumed in their natural diets.

Trends in cortisol and molecular biomarker data corresponded to both survival and feeding. A non-significant but detectable elevation in cortisol levels at 5, 12 and 25 NTU suggests higher stress in these treatments. As discussed above, at the lower turbidity levels (5 and 12 NTU), light sensitivity along with the confined water depth of the exposure vessel may have played important roles. Turbidity ranges between 35 and 80 NTU resulted in lower levels of cortisol in late-larval delta smelt. These trends support what was observed in the survival and the feeding response, indicating a similar range as favourable. Reduced cortisol levels observed at the highest turbidity (250 NTU) are likely to be associated with impairment of respiration; elevated mortality resulted in these treatments. The highest turbidity level (250 NTU) unexpectedly resulted in low cortisol levels, but gene expression responded significantly [e.g. *gst* indicative of oxidative and osmotic stress (respiration), *hsp70* involved in general stress and *glut2* involved in energy metabolism]. Taken together, this suggests that turbidities above 120 NTU do not necessarily evoke a primary stress response after 24 h, but secondary stress responses are at play, as indicated by the molecular biomarkers.

Increased stress also affects a number of organismal parameters owing to reallocation of metabolic energy. When stressed organisms spend more energy on regaining homeostasis (e.g. respiration, locomotion, tissue repair and hydromineral regulation), less energy can be invested in development and growth. Reduction in growth can in turn lead to starvation, reduced fitness and reduced activity and, ultimately, death (Wendelaar Bonga, 2011). Reduced activity and fitness have further implications on predator avoidance, as well as the capture of prey items. Stress is also known to affect the susceptibility to disease (Snieszko, 1974; Pickering and Pottinger, 1989), and cortisol has been demonstrated to function as a key mediator modulating the immune response (Tort, 2011). Reduced survival in larval life stages owing to elevated or decreased turbidities can have significant long-term impacts on reproduction of the declining delta smelt population. In particular, low numbers of young fish will lead to a diminished population size and low numbers of adults potentially migrating back to the spawning grounds to secure the continued existence of this species. In addition, this might further reduce the genetic diversity and exacerbate the documented population bottleneck for delta smelt (Fisch *et al*., 2011).

Compared with the juvenile life stage (Hasenbein *et al*., 2013), the larval life stage appears to have more limited tolerance ranges for turbidity. Previous studies with juvenile delta smelt (120 dph) showed enhanced feeding at low turbidities and constant feeding up to 120 NTU, with a strong decline in feeding at 250 NTU (Hasenbein *et al*., 2013). In juvenile fish, stress-related biomarkers were largely unresponsive to turbidities up to 120 NTU but showed very high expression at 250 NTU (Hasenbein *et al*., 2013). In contrast, for the larval life stage, significant adverse effects in reduced feeding and elevated expression below 12 NTU and above 80 NTU were determined. Taken together, these data indicate that tolerance ranges are life-stage dependent and that ontogenetic changes need to be taken into consideration in tolerance range and niche assessments. Differential tolerances to environmental parameters, such as temperature and salinity, between life stages of delta smelt have also been observed in other studies (e.g. Komoroske *et al*., 2014, 2015). Thus, understanding the tolerance ranges and niche dimensions for each life stage of a species is of utmost importance in order to conduct and pursue effective conservation efforts.

All study endpoints (survival, feeding, biochemical and molecular stress indicators) delivered consistent results, providing confidence to our estimate of the larval delta smelt turbidity requirements. However, some could argue that measuring only a couple of endpoints, such as survival and food intake, is easier and more cost effective to assess, whereas recent reviews have highlighted the importance of a mechanistic understanding of physiological responses, in particular with respect to informed management decisions (Geist, 2015; Horodysky *et al*., 2015). In general, when measuring endpoints across different biological levels, the correspondence is not always certain. Thus it is important to determine multiple, integrative endpoints at different levels of biological organization first, in order to make meaningful interpretations before moving forward with use of simple or fewer metrics. The approach used in the present study expands on the approach used by Newcombe and Macdonald (1991), by also including the molecular level and by more generally considering turbidity as part of the ecological niche of a species (taking both positive and negative effects into account). For instance, measuring molecular biomarkers involved in several different metabolic functions, such as the stress response (hypothalamic–pituitary–interrenal axis), energy metabolism, generalized stress and osmotic stress, reveals underlying physiological mechanisms and helps to evaluate potential adverse outcomes caused by stressors. Together with cortisol, molecular biomarkers provide explanations for behavioural and whole-organism endpoints, such as feeding and survival. In addition, some adverse effects would not be detected when solely measuring whole-organism endpoints. The use of multiple-level endpoints is transferable to numerous fish species and stressor types, illustrating their potential for wide use in the field of conservation physiology. Even though these results clearly show that the early life stage of the delta smelt is affected by changes in turbidity, it should be emphasized that these experiments were relatively short term and tested a single turbidity material, light intensity, prey item type and life stage. Further tests that integrate these variables to a broader extent are required to explore more comprehensively the effects of turbidity on the delta smelt.

## Supplementary material

Supplementary material is available at *Conservation Physiology* online.

## Funding

Funding was provided by the US Department of Interior, Bureau of Reclamation (contract R12AP20018 to R.E.C. and N.A.F.), the California Delta Stewardship Council (contract 201015533 to R.E.C. and N.A.F.), the State and Federal Contractors Water Agency (contract no. 15-13 to R.E.C.) and the University of California Agricultural Experiment Station (grant number 2098-H to N.A.F.). Partial student funding was provided to M.H. by the Bavarian Elite Programme Universität Bayern e.V.—Scholarship for graduate and post-graduate students and to L.M.K. by the National Science Foundation Graduate-12 Fellowship Program (under the Division of Graduate Education grant number 0841297 to S. L. Williams and B. Ludaescher) and the California Sea Grant Delta Science Doctoral Fellowship (R/SF-56). The authors acknowledge the support of the Technische Universität München Graduate School's Faculty Graduate Center Weihenstephan at Technische Universität München, Germany.

## Supplementary Material

Supplementary Data
