# Supplementary material for: Assessments at multiple levels of biological organization allow for an integrative determination of physiological tolerances to turbidity in an endangered fish species
Source: Conserv Physiol. 2016 Mar 16;4(1):cow004. doi: 10.1093/conphys/cow004 (PMC4795446; doi:10.1093/conphys/cow004)
Supplement: Supplementary Data [file cow004supp.zip › cow004supp.docx]

**Table S1, Supplementary.** Detailed statistical information on individual ANOVA models and Kruskal- Wallis Test for the effect turbidity for endpoints survival, feeding, cortisol and each gene of the qPCR data set, respectively.

| **Anova Model Parameter** | **Effect** | **Sum Sq** | **DF** | **Mean Sq** | **F value** | **Pr(>F)** |
| --- | --- | --- | --- | --- | --- | --- |
| Survival | Turbidity | 4118 | 7 | 588.3 | 7.862 | 0.0000689 |
| Feeding | Turbidity | 1287 | 7 | 183.9 | 3.12 | 0.0182 |
| Cortisol | Turbidity | 15.34 | 7 | 2.191 | 1.684 | 0.144 |
| HSP70 | Turbidity | 5.878 | 7 | 0.8398 | 6.012 | 0.000037 |
| GLUT 2 | Turbidity | 10.55 | 7 | 1.5079 | 2.797 | 0.0151 |
| NH4+ trans | Turbidity | 14.86 | 7 | 2.123 | 3.97 | 0.00152 |
| GST | Turbidity | 8.74 | 7 | 1.25 | 2.66 | 0.02 |
| Catalase | Turbidity | 2.612 | 7 | 0.3731 | 1.3 | 0.269 |
| IGF | Turbidity | 2.406 | 7 | 0.3437 | 0.837 | 0.562 |
| NF-kB | Turbidity | 2.654 | 7 | 0.3792 | 1.296 | 0.271 |
| HIF1a | Turbidity | 3.80 | 7 | 0.54 | 0.84 | 0.56 |
| 11-Beta-HSD-2 | Turbidity | 2.54 | 7 | 0.36 | 0.64 | 0.72 |
| MR1 | Turbidity | 3.40 | 7 | 0.49 | 1.31 | 0.26 |
| GR2 | Turbidity | 2.29 | 7 | 0.33 | 0.70 | 0.68 |
| Na/K ATPase | Turbidity | 4.49 | 7 | 0.64 | 1.87 | 0.09 |
| **Kruskal Wallis Test** | **Effect** | **Chi Squared** | **DF** |  |  | **Pr(>F)** |
| 11-Beta-HSD-1 | Turbidity | 5.43 | 7 |  |  | 0.61 |
| POMC | Turbidity | 1.75 | 7 |  |  | 0.97 |
| SGK3 | Turbidity | 5.66 | 7 |  |  | 0.5795 |

Abbreviations: DF: Degrees of Freedom, Sum Sq: Sum of squares, Mean Sq: Mean of squares, HSP70: Heat shock Protein 70kD, GLUT 2: Glucose Transporter 2, NH4+ trans: Ammonium transporter, GST: Glutathione-S-Transferase, IGF: Insulin like growth factor, NF-kB, Nuclear factor k-Beta, HIF1a: Hypoxia inducible factor 1 alpha, 11-Beta-HSD-1: 11-β-Hydroxysteroid-Dehydrogenase-Type 1, 11-Beta-HSD-2: 11-β-Hydroxysteroid-Dehydrogenase-Type 2, MR1: Mineralocorticoid receptor 1, GR2: Glucocorticoid receptor 2, POMC: Pro-Opiomelanocortin, SGK3: Serum/Glucocorticoid regulated kinase 3, Na/K ATPase: Sodium Potassium ATPase.

**Table S2, Supplementary**. Fold-change in Gene Transcription for all genes that were not statistically significant. Values above 1.0 indicate upregulated genes and value below 1.0 indicate down-regulated genes.

| **Turbidity** |  |  | **IGF** | **NF-KB** | **HIF 1a** | **11-Beta-HSD-1** | **11-Beta-HSD-2** | **MR 1** | **GR2** | **POMC** | **SGK3** | **Na/K ATPase** | **Catalase** |
| --- | --- | --- | --- | --- | --- | --- | --- | --- | --- | --- | --- | --- | --- |
| **5** |  | Average | 1.83 | 2.41 | 0.98 | 2.68 | 1.33 | 2.71 | 3.01 | 1.12 | 0.61 | 1.33 | 1.48 |
|  |  | SE | 0.30 | 0.41 | 0.19 | 0.47 | 0.27 | 0.53 | 0.85 | 0.44 | 0.10 | 0.23 | 0.13 |
| **12** |  | Average | 1.67 | 2.13 | 1.11 | 2.34 | 0.92 | 2.38 | 2.03 | 1.07 | 0.49 | 1.08 | 1.55 |
|  |  | SE | 0.28 | 0.23 | 0.20 | 0.43 | 0.17 | 0.33 | 0.32 | 0.31 | 0.10 | 0.21 | 0.32 |
| **25** |  | Average | 1.62 | 2.48 | 1.30 | 2.02 | 1.33 | 2.10 | 1.69 | 0.66 | 0.67 | 1.04 | 1.57 |
|  |  | SE | 0.39 | 0.37 | 0.32 | 0.38 | 0.26 | 0.35 | 0.21 | 0.17 | 0.16 | 0.21 | 0.25 |
| **35** |  | Average | 2.46 | 2.76 | 1.35 | 2.46 | 1.58 | 2.34 | 2.30 | 0.88 | 1.41 | 1.21 | 1.88 |
|  |  | SE | 0.54 | 0.47 | 0.28 | 0.39 | 0.34 | 0.46 | 0.35 | 0.16 | 0.76 | 0.13 | 0.26 |
| **50** |  | Average | 2.71 | 3.06 | 1.95 | 2.60 | 1.21 | 3.23 | 2.51 | 0.94 | 0.95 | 2.20 | 2.44 |
|  |  | SE | 0.41 | 0.30 | 0.33 | 0.32 | 0.17 | 0.38 | 0.28 | 0.17 | 0.16 | 0.26 | 0.30 |
| **80** |  | Average | 2.28 | 2.97 | 1.72 | 2.42 | 1.58 | 2.60 | 2.59 | 0.95 | 0.80 | 1.60 | 2.13 |
|  |  | SE | 0.33 | 0.30 | 0.38 | 0.61 | 0.16 | 0.35 | 0.42 | 0.20 | 0.08 | 0.09 | 0.22 |
| **120** |  | Average | 2.49 | 3.34 | 1.58 | 3.28 | 1.56 | 3.80 | 3.78 | 1.87 | 0.99 | 1.78 | 2.58 |
|  |  | SE | 0.46 | 0.41 | 0.28 | 0.53 | 0.15 | 0.72 | 0.80 | 0.99 | 0.17 | 0.14 | 0.30 |
| **250** |  | Average | 2.69 | 3.61 | 1.43 | 2.84 | 1.17 | 3.15 | 2.04 | 0.97 | 0.71 | 1.62 | 2.37 |
|  |  | SE | 0.45 | 0.38 | 0.26 | 0.37 | 0.23 | 0.57 | 0.39 | 0.22 | 0.12 | 0.29 | 0.51 |

Abbreviations: IGF: Insulin like growth factor, NF-kB: Nuclear factor k-Beta, HIF1a: Hypoxia inducible factor 1 alpha, 11-Beta-HSD-1: 11-β-Hydroxysteroid-Dehydrogenase-Type 1, 11-Beta-HSD-2: 11-β-Hydroxysteroid-Dehydrogenase-Type 2, MR1: Mineralocorticoid receptor 1, GR2: Glucocorticoid receptor 2, POMC: Pro-Opiomelanocortin, SGK3: Serum/Glucocorticoid regulated kinase 3, Na/K ATPase: Sodium Potassium ATPase, SE: Standard Error.
